# Metabolic profiling by gas chromatography-mass spectrometry of energy metabolism in high-fat diet-fed obese mice

**DOI:** 10.1371/journal.pone.0177953

**Published:** 2017-05-16

**Authors:** Daxesh P. Patel, Kristopher W. Krausz, Cen Xie, Diren Beyoğlu, Frank J. Gonzalez, Jeffrey R. Idle

**Affiliations:** 1 Laboratory of Metabolism, Center for Cancer Research, National Cancer Institute, National Institutes of Health, Bethesda, MD, United States of America; 2 Hepatology Research Group, Department of Clinical Research, University of Bern, Bern, Switzerland; University of Nebraska Medical Center, UNITED STATES

## Abstract

A novel, selective and sensitive single-ion monitoring (SIM) gas chromatography-mass spectrometry (GCMS) method was developed and validated for the determination of energy metabolites related to glycolysis, the tricarboxylic acid (TCA) cycle, glutaminolysis, and fatty acid β-oxidation. This assay used *N-tert*-butyldimethylsilyl-*N*-methyltrifluoroacetamide (MTBSTFA) containing 1% *tert*-butyldimethylchlorosilane (TBDMCS) as derivatizing reagent and was highly reproducible, sensitive, specific and robust. The assay was used to analyze liver tissue and serum from C57BL/6N obese mice fed a high-fat diet (HFD) and C57BL/6N mice fed normal chow for 8 weeks. HFD-fed mice serum displayed statistically significantly reduced concentrations of pyruvate, citrate, succinate, fumarate, and 2-oxoglutarate, with an elevated concentration of pantothenic acid. In liver tissue, HFD-fed mice exhibited depressed levels of glycolysis end-products pyruvate and lactate, glutamate, and the TCA cycle intermediates citrate, succinate, fumarate, malate, and oxaloacetate. Pantothenate levels were 3-fold elevated accompanied by a modest increased gene expression of *Scl5a6* that encodes the pantothenate transporter SLC5A6. Since both glucose and fatty acids inhibit coenzyme A synthesis from pantothenate, it was concluded that these data were consistent with downregulated fatty acid β-oxidation, glutaminolysis, glycolysis, and TCA cycle activity, due to impaired anaplerosis. The novel SIM GCMS assay provided new insights into metabolic effects of HFD in mice.

## Introduction

In recent years, obesity has become a serious worldwide health concern [1]. Chronic diseases like cancer, cardiovascular disease, steatohepatitis and type 2 diabetes mellitus (insulin resistance) are commonly associated with obesity [2]. Complex interactions of both genetic and environmental factors with excessive fat accumulation are responsible for obesity development [3–5]. There is no safe and effective drug therapy as obesity due to a chronic imbalance between energy intake and expenditure [6]. Therefore, mechanistic aspects of obesity development have become the focus of many investigations, specifically using system biological approaches.

Omics studies revealed that the development of obesity is accompanied by changes in multiple metabolic pathways such as the TCA cycle, fatty acid redox metabolism and glycolysis [4,7–9]. Genomic and transcriptomic studies revealed that a number of genes are associated with common human obesity development, with their functions involving hormone regulations and insulin signaling, energy homeostasis, lipogenesis [7], nicotinamide phosphorylation and inflammation and fatty acid β-oxidation in rodents [7]. Proteomic studies revealed that obesity is associated with significant differential expression of proteins in adipose tissue, muscle and liver [8], and serum [10] and alterations involving mitochondrial, cytoskeletal and structural proteins together with TCP1 complex proteins [9].

Metabolomic studies have been reported to explore the obesity effects on HFD-induced obese rodents [11–13] and for human obesity [10,14]. These studies revealed that obese mammals have clearly different phenotypes and metabotypes of the metabolism of fatty acids, amino acids, acylcholines, as well as for glycolysis and the TCA cycle [10–22]. Moreover, the gut microbiota have significant roles to play in HFD-induced obesity in terms of energy harvest [3,23], insulin resistance [24], and modulations to host metabolisms [25,26]. However, these previous studies provided limited information on the dynamic metabolic changes associated with the development of obesity they mostly focused on the consequences while the dynamic processes of obesity development remain to be revealed.

The HFD-induced mouse obesity model resembles human obesity in phenotype and in its complications [27]. Therefore, the role of TCA cycle intermediates, fatty acids, amino acids and other metabolism associated pathways in obesity development may be examined in HFD-fed mice using targeted metabolomics. Targeted metabolomics detects and quantitates variations in endogenous and exogenous metabolite composition for an integrated biological system. Typically, GCMS, liquid chromatography-tandem mass spectrometry (LCMS/MS), and nuclear magnetic response (NMR) are employed. These methodologies are useful to explain the detailed metabolic adaptations associated with obesity development and have been successfully applied to reveal the biochemical aspects of metabolic disorders [28,29].

In this study, we sought to understand if the energy metabolites involved in glycolysis, the TCA cycle, glutaminolysis, and fatty acid β-oxidation were altered in C57BL/6J obese mice that had been fed a HFD. To accomplish this, a novel, specific and sensitive GCMS assay for energy metabolites was developed that covered 13 TCA cycle and accessory metabolites.

## Materials and methods

### Animal studies and sample collection

**Fig 1:**
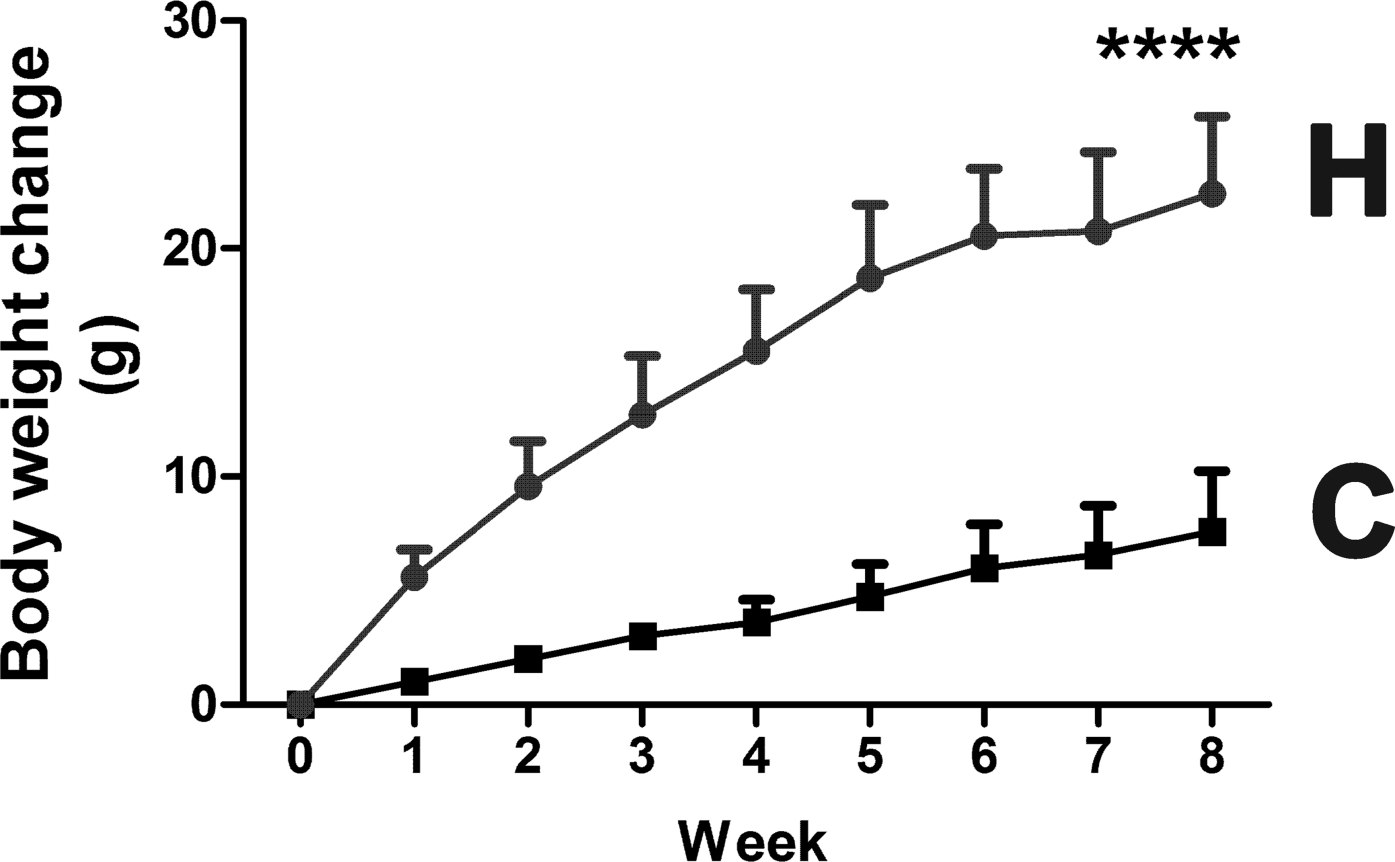
Body weight gain for mice fed HFD diet and control chow over 8 weeks. **** p < 0.0001.

Mouse experimental procedures, performed according to the National Institutes of Health guidelines, were reviewed and approved by the National Cancer Institute Animal Care and Use Committee. Mice were treated humanely and with regard for the alleviation of suffering. Male 6- to 8-week-old mice on a C57BL/6N background were purchased from Charles River Laboratories (Wilmington, MA) and individually housed in their home cages in a specific pathogen-free environment controlled for temperature and light (25°C, 12h light: 12h dark cycle), and humidity (45–65%), with *ad libitum* access to water and pelleted NIH-31 chow. All mice were randomly assigned to experimental groups. Study group and control group mice showed no difference in body weight gain before treatment. Obesity was induced in male 6–8 week-old wild-type mice (study group) by feeding a HFD (60% Kcal from fat; Bio-Serv, Flemington, NJ) or NIH-31 diet (control group) for 8 weeks. Mice were weighed at weekly intervals and the HFD-fed mice gained three-times more body weight than the control chow-fed mice (Fig 1). At the end of the study, all animals were fasted for 12 h prior to being euthanized by CO_2_ asphyxiation. Blood samples and liver were collected at the end of study from each group for metabolic analysis. All remaining samples were snap-frozen using liquid nitrogen immediately after collection and stored at -80°C until further analysis.

### Chemicals and materials

All metabolite standards (> 98%) and D,L-norleucine (DLN; internal standard) (> 99%) were purchased from Sigma-Aldrich (St. Louis, MO). The derivatization reagent MTBSTFA + 1% TBDMCS (*N*-*tert*-butyldimethylsilyl-*N*-methyltrifluoroacetamide with 1% *tert*-butyldimethyl chlorosilane) was purchased from Regis Technologies Inc. (Morton Grove, IL). BSTFA (*N*,*O*-bis(trimethylsilyl)trifluoroacetamide) was purchased from Sigma-Aldrich. LCMS grade acetonitrile was purchased from Fisher Scientific (Waltham, MA). Deionized water was obtained from Milli-Q water purification system from EMD Millipore (Billerica, MA).

### Chromatographic and mass detection parameters

Silylated derivatives of energy metabolites were separated on a capillary column (30 m × 0.250 mm, 0.25 μm; Agilent Technologies, Foster City, CA). Analyses were performed with an Agilent 6890N gas chromatograph coupled to an Agilent 5973 mass-selective detector (MSD) with following chromatographic conditions: Initial temperature 50°C for 2 min, increasing to 150°C at 20°C/min over 5 min and finally to 300°C at 8°C/min for 20 min. The front inlet temperature was 250°C operating with a split ratio of 1:25. MSD ion source and interface temperature was 280°C. The MSD operated in EI mode at 70 eV. SIM mode of 30–650 m/z was used for the analyses. Carrier gas was He (1.0 ml/min). GCMS data were acquired and processed using Agilent MassHunter WorkStation software.

### Calibration standards and quality control samples

Standard stock solutions of energy metabolites (5.0 mM) were prepared in acetonitrile. Further, working solutions were prepared using intermediate solutions of 500.0 μM and 50.0 μM in acetonitrile:water (50:50 v/v). Calibration curve standards were made at 0.5, 2.0, 5.0, 10, 25, 50 μM, while the quality control samples were prepared at four levels, that is, 40 μM (HQC, high quality control), 20 μM (MQC, middle quality control), 7.5 μM (LQC, low quality control), 0.5 μM (LLOQ QC, lower limit of quantitation quality control). A stock solution of the internal standard (2.0 mM, DLN) was used. Further, working solutions were prepared from the stock solution in acetonitrile:water (50:50 v/v) at 10.0 μM for DLN.

### Preparation of liver samples

C57BL/6N mouse liver tissue 20 ± 0.05 mg (chow and HFD diet-fed) was placed in 0.7 ml 70% acetonitrile:water (70:30 v/v) and 20 μl of DLN (IS,10μM) added, followed by homogenization using a Precellys homogenizer (Bertin Instruments, Montigny-le-Bretonneux, France), utilizing 1.0 mm zirconia/silica beads for 30 sec at 6500 rpm. The samples were centrifuged at 20,000g for 10 min at 4°C and 600 μl of supernatant was taken and dried in a SpeedVac concentrator at room temperature. The dried residue was derivatized by adding 50 μl MTBSTFA + 1% TBDMCS, and sonicated for 30 min at room temperature. The samples were diluted with 50 μμl acetonitrile, briefly vortexed for 10 s and 1.00 μl was injected into the GCMS using an autosampler. It was found that neither isocitric acid nor pantothenic acid derivatized well with MTBSTFA + TBDMCS and therefore these compounds were determined in liver as above, only using BSTFA (50 μl) with 30 min sonication at room temperature. After dilution with 50 μl acetonitrile, samples were briefly vortexed for 10 s and 1.00 μl analyzed as above by GCMS.

### Preparation of serum samples

To an aliquot of 50 μl serum, 20 μl of DLN (10μM) was added, the sample vortexed for 10 s, and 0.7 ml of acetonitrile added and vortex mixed for a further 1 min. The samples were centrifuged at 20,000g for 10 min at 4°C and 0.6 ml supernatant transferred to 2 ml vials. The samples were dried in a SpeedVac concentrator at room temperature and the dried residue derivatized with using 50 μl MTBSTFA + 1% TBDMCS. The samples were sonicated for 30 min at room temperature and diluted with 50 μl acetonitrile, briefly vortexed for 10 s and 1.00 μl was injected into the GCMS using an autosampler. For the analysis of isocitric acid and pantothenic acid, dried residues were derivatized as for liver tissue extracts using *N*,*O*-bis(trimethylsilyl)trifluoroacetamide (BSTFA).

### Validation procedures

Three calibration curves were plotted covering the range of 0.05–50μM for TCA intermediates using least squares regression and 1/*x*^*2*^ as a weighting factor. The area response ratio for analyte/IS obtained from single ion monitoring was used for regression analysis. The acceptance criterion for a calibration curve was a correlation coefficient (r^2^) ≥ 0.99 and the lowest standard on the calibration curve was accepted as the assay sensitivity expressed as LLOQ. Intra-batch accuracy and precision was determined by analyzing six replicates of QC samples along with calibration curve standards on the same day, while the inter-batch accuracy and precision were assessed by analyzing three precision and accuracy batches on three consecutive days. The precision (% CV) at each concentration level from the nominal concentration was expected to be not greater than 15% and the accuracy to be within ±15% as per USFDA guidelines [30], except for the LLOQ where it can be 80–120% of the nominal concentration. Reinjection reproducibility was also checked by re-injecting one entire validation batch.

Stability tests were conducted for stock solutions of analytes and ISs for short term and long term stability at 18°C and 4°C respectively. All stability results for spiked samples were evaluated by measuring the area response ratio (analyte/IS) of stability samples against freshly prepared comparison standards. QC samples at HQC and LQC levels were prepared to check for bench top, autosampler (wet extract), processed sample, dry extract, freeze-thaw and long term (-80°C) stability. The acceptance criterion was ± 10.0% deviation (from the nominal value) for stock solutions and ± 15% deviation for all other storage conditions.

### RNA extraction and qPCR analysis

mRNA was prepared from frozen mouse liver as described [31]. Gene expression for *Slc5a6* and *Gapdh* was analyzed by qPCR using SYBR® GreenER™ Reagent System (Invitrogen, Carlsbad, CA) in a 7900 HT Fast Real-Time PCR system (Applied Biosystems, Carlsbad, CA). Relative expression calculated by the ΔΔCt method using *Gapdh* mRNA as the internal control, and statistical analyses were performed using the ΔCt values. Primer sequences for gene expression analyses are available on request.

### Statistical analysis

Energy metabolites were estimated by non-compartmental analysis using MassHunter Workstation Software Quantitative Analysis Version B.05.01 (Agilent Technologies). Group differences were evaluated with a nonparametric two-tailed Mann-Whitney *U* test using GraphPad Prism 6 (San Diego, CA). Experimental values are presented as mean ± S.D.

## Results

### GCMS assay development

**Fig 2:**
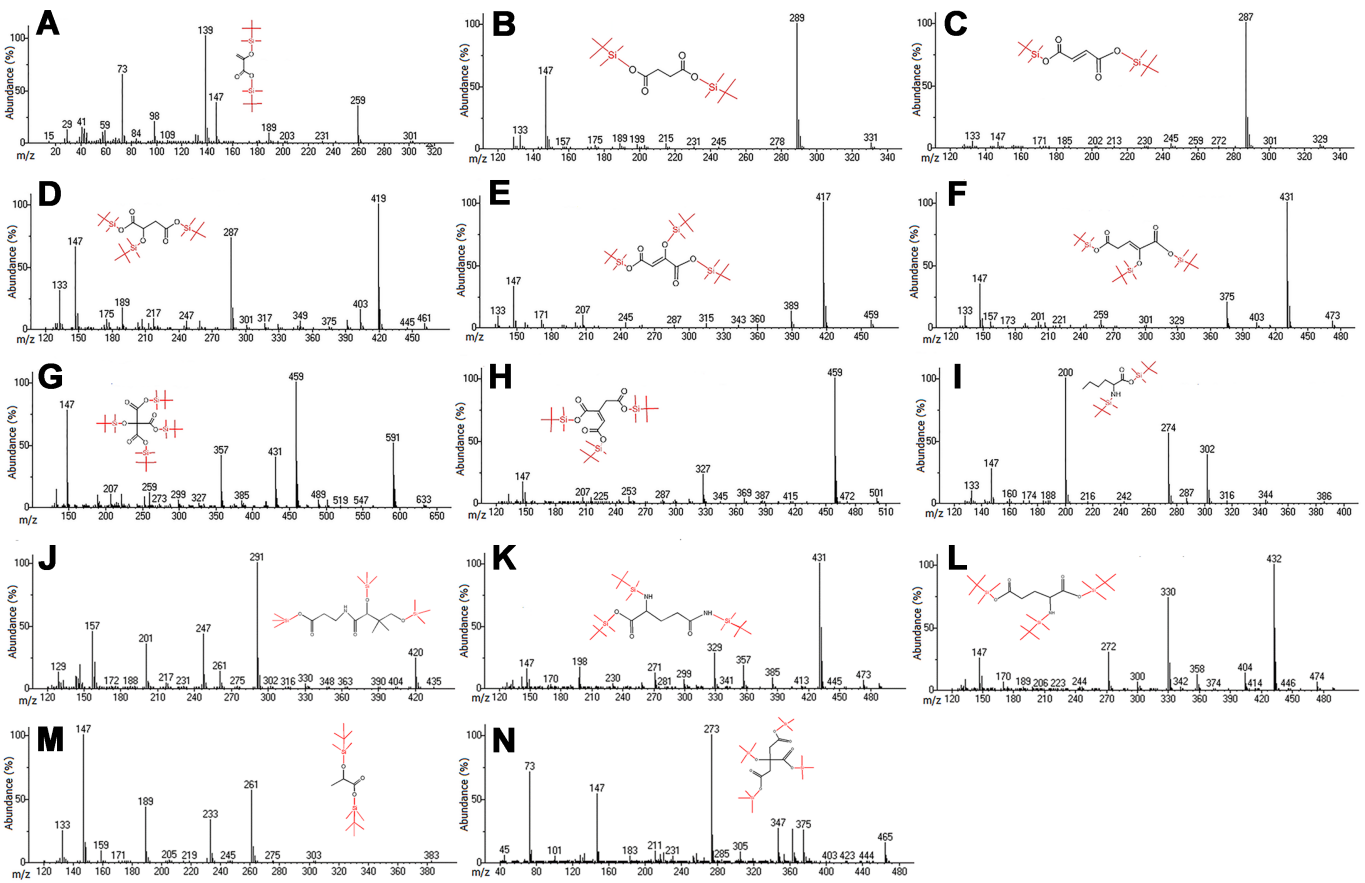
Mass spectra showing target and qualifier ions for a) pyruvic acid, b) succinic acid, c) fumaric acid, d) malic acid, e) oxaloacetic acid, f) 2-oxoglutaric acid, g) citric acid, h) *cis*-aconitic acid, i) DL-norleucine (IS), j) pantothenic acid, k) glutamine, l) glutamic acid, m) lactic acid, and n) isocitric acid. The metabolites j) and n) were derivatized with BSTFA, rather than MTBSTFA + 1% TBDMCS.

Published methods for the simultaneously determination of energy metabolites using GCMS, LC-MS/MS or NMR suffer from either cumbersome extraction procedures, long retention times or low sensitivity. The first aim of this study was to develop a rugged GCMS method that offered the combined advantage of sensitivity, selectively, simplicity of extraction procedure, and high throughput, adequately controlled for potential errors during extraction and analysis, thereby ensuring accuracy of the generated data. Mass spectrometry parameters were optimized to maximize the response for the energy metabolites and IS represented in Table 1. The full scan spectra showed consistent and predominant target and qualifier ions for energy metabolites with NIST mass spectral library matches (86–99%) as shown in Table 1. The electron ionization mass spectra under optimized conditions for the TCA metabolites and IS are presented in Fig 2. The most stable and consistent fragment ions for the TBDMS derivatives of pyruvic acid, citric acid, *cis*-aconitic acid, 2-oxoglutaric acid, succinic acid, fumaric acid, malic acid, oxaloacetic acid, lactic acid, glutamine, glutamic acid, isocitric acid, pantothenic acid, and DLN (IS) were observed at *m/z* 139, 459, 459, 431, 289, 287, 419, 417, 147, 73, 73, 273 (TMS derivative), 73 (TMS derivative), and 200, respectively.

**Table 1:** Quantitation by GCMS of energy metabolites.

Energy metabolite	Target ion (m/z)	Qualifier ions (m/z)	Retention time (min)	Match % (NIST Library)	Limit of detection (μM)	System suitability (%CV) (n = 6)
Pyruvic acid	139	259,189	10.1	86	0.03	1.18
Citric acid	459	357,591	22.7	99	0.20	1.45
*Cis*-aconitic acid	459	327,501	20.2	97	0.03	2.09
2-Oxoglutaric acid	431	375,473	18.7	98	0.20	3.92
Succinic acid	289	278,331	12.8	99	0.01	2.09
Fumaric acid	287	301,329	13.2	99	0.03	1.76
Malic acid	419	287,461	17.2	99	0.03	2.64
Oxaloacetic acid	417	389,459	17.4	91	0.01	4.42
Lactic acid	261	189,233	9.7	99	0.01	3.69
Glutamine	431	329,357	20.8	98	0.03	4.02
Glutamic acid	432	330,272	19.1	98	0.03	4.56
Isocitric acid	273	363,465	14.6	92	0.20	4.98
Pantothenic acid	291	201,247	15.9	94	0.03	3.84
D,L-Norleucine (IS)	200	274,302	12.7	99	—	3.69

%CV: Percent coefficient of variance.

In the present study, several trials were carried out on tissue and serum with different extraction techniques, namely protein precipitation (PP) and liquid-liquid extraction (LLE) followed by use of different silylation reagents. The serum PP was extracted using methanol and acetonitrile as protein precipitants; however, with methanol the recovery showed poor chromatography with high variability of the IS (50–70%) at LLOQ and LQC levels for malic acid, oxaloacetic acid and 2-oxoglutaric acid. Thus, acetonitrile was used for protein precipitation before derivatization of the energy metabolites. Further, LLE was tested for liver tissue samples with different organic diluents using dichloromethane, ethyl acetate, chloroform, *n*-hexane, alone and in combination under neutral and alkaline conditions. The results showed very poor chromatography with high IS variability (65–80%) in almost all the solvents, especially for LLOQ and LQC samples as most of the energy metabolites are highly water soluble. Thus, monophasic liquid extraction was carried out on using 70% aqueous acetonitrile to overcome the problems encountered during LLE.

Previous methods have used *N*,*O*-bis-(trimethylsilyl)acetamide (BSA) [32], *N*-trimethylsilyl-*N*-methyl trifluoroacetamide (MSTFA) [33], BSTFA and trimethylchlorosilane (TMCS) [34], tributylamine [35], derivatizing reagents with a longer analysis time [35] and lower sensitivity [34] for selected energy metabolites [33,36] analyzed by GCMS [34,36] and LCMS [33,35] (S1 Table). A major lacuna in even the most recently published methods is metabolite coverage. Our method quantitates 13 TCA and accessory metabolites (Table 2), while the most recent reports using BSTFA + 1% TCMS quantitated only 7/13 metabolites [37], and 6/13 metabolites [38], and using MSTFA quantitated only 3/13 metabolites [39]. This meager metabolite coverage in recent published reports was a major factor in our assay development. In addition, many published methods took an overly long time for the separation of analytes under gradient elution and thus may not be useful for high-throughput analysis. Therefore, different silylation reagents were evaluated, including, *tert*-butyldimethylsilyl (TBDMS), BSTFA, MSTFA, and MTBSTFA + 1% TBDMCS. Among these, good chromatography was observed and all energy metabolites could be quantitated with MTBSTFA + 1% TBDMCS, except for isocitric acid and pantothenic acid. However, BSTFA was able to generate a stable silyl derivative for these two metabolites. The principal limitation of this method was the failure to separate citric and isocitric acids on the column used. Nevertheless, MTBSTFA + 1% TBDMCS was chosen as the derivatization reagent for the determination of energy metabolites in serum and liver tissues because it gave better separation of analytes, with more stable derivatives, which yielded characteristic [M-57]^+^ fragment ions [40,41] with a superior performance than BSTFA for non-sterically-hindered substrates [41]. Table 2 shows the response ratios for each of the energy metabolites determined in mouse serum and liver tissues after feeding HFD and normal chow diet.

**Table 2:** The results of targeted TCA intermediates in serum and liver.

Energy metabolite	Derivatization reagent	Area response ratio ± S.D. (Liver, n = 5)		Area response ration ± S.D (Serum, n = 5)	
		HFD	CHOW	HFD	CHOW
Pyruvic acid	MTBSTFA + 1%TBDMCS	0.013 ± 0.010	0.049 ± 0.019	1.22 x 10^−4^± 5.68 x 10^−5^	2.39 x 10^−4^ ± 3.24 x 10^−5^
Citric acid	MTBSTFA + 1%TBDMCS	0.095 ± 0.100	0.293 ± 0.117	1.96 x 10^−3^± 4.30 x 10^−4^	2.92 x 10^−3^± 4.58 x 10^−4^
Cis-aconitic acid	MTBSTFA + 1%TBDMCS	0.027 ± 0.014	0.043 ± 0.014	1.91 x 10^−4^± 1.42 x 10^−4^	4.47 x 10^−4^± 2.63 x 10^−4^
2-Oxoglutaric acid	MTBSTFA + 1%TBDMCS	0.105 ± 0.031	0.221 ± 0.144	4.61 x 10^−4^± 1.36 x 10^−4^	1.20 x 10^−3^± 4.00 x 10^−4^
Succinic acid	MTBSTFA + 1%TBDMCS	5.473 ± 2.041	17.981 ± 9.505	0.012 ± 0.002	0.024 ± 0.10
Fumaric acid	MTBSTFA + 1%TBDMCS	0.364 ± 0.489	2.543 ± 0.554	4.92 x 10^−3^± 2.31 x 10^−3^	7.84 x 10^−3^± 2.85 x 10^−3^
Malic acid	MTBSTFA + 1%TBDMCS	0.012 ± 0.005	0.946 ± 0.435	1.84 x 10^−3^± 2.97 x 10^−4^	2.82 x 10^−3^± 9.01 x 10^−4^
Oxaloacetic acid	MTBSTFA + 1%TBDMCS	0.022 ± 0.016	0.119 ± 0.048	ND	ND
Lactic acid	MTBSTFA + 1%TBDMCS	3.149 ± 1.114	7.833 ± 2.376	0.894 ± 0.457	1.194 ± 0.276
Glutamine	MTBSTFA + 1%TBDMCS	0.004 ± 0.004	0.004 ± 0.001	0.060 ± 0.011	0.027 ± 0.008
Glutamic acid	MTBSTFA + 1%TBDMCS	0.257 ± 0.054	0.425 ± 0.090	ND	ND
Isocitric acid	BSTFA	0.399 ± 0.084	0.616 ± 0.125	3.138 ± 0.643	3.732 ± 0.435
Pantothenic acid	BSTFA	0.145 ± 0.058	0.052 ± 0.012	0.602 ± 0.051	0.462 ± 0.035

**Fig 3:**
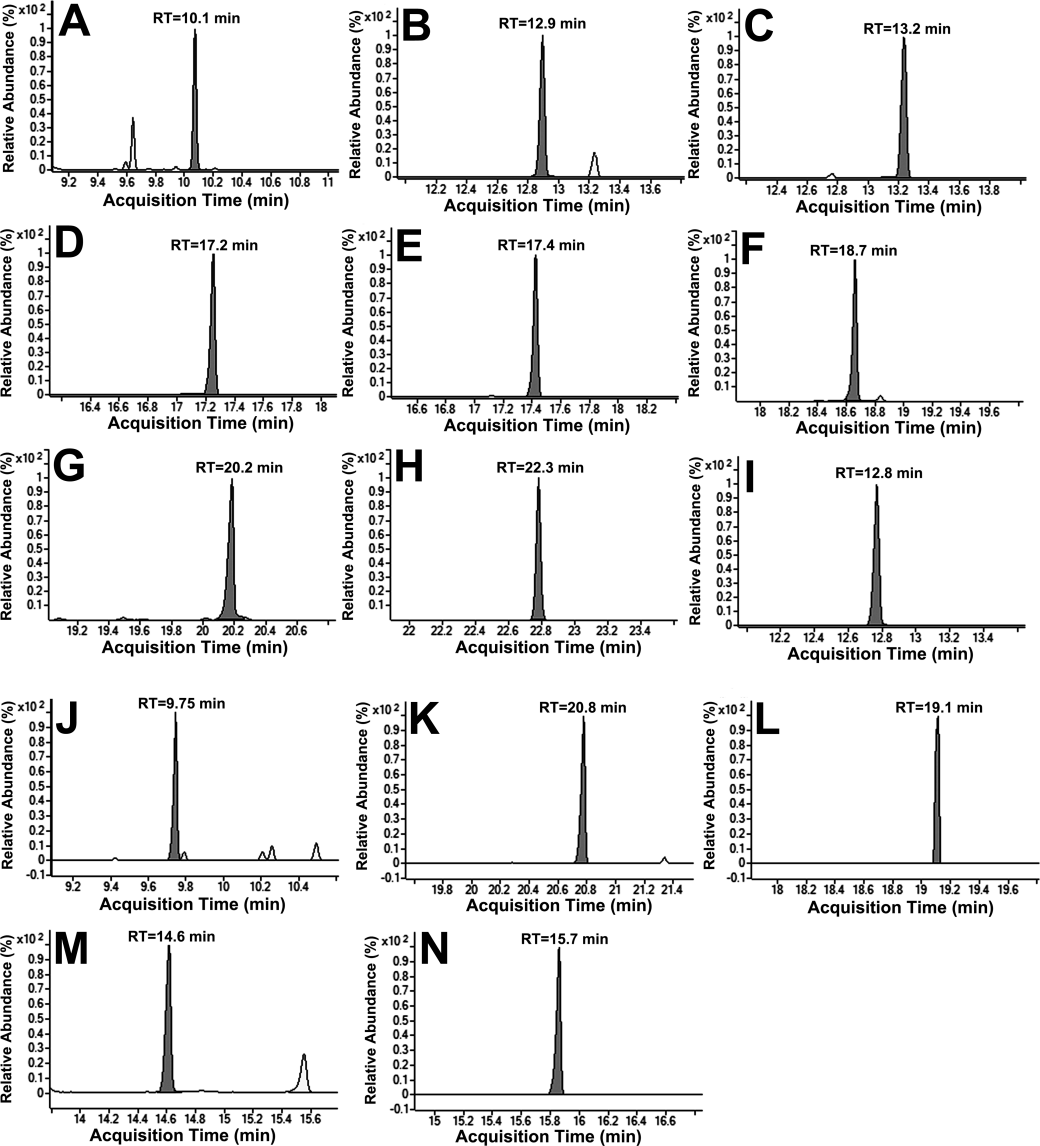
Representative single ion monitoring chromatograms of the energy metabolites. a) pyruvic acid, b) succinic acid, c) fumaric acid, d) malic acid, e) oxaloacetic acid, f) 2-oxoglutaric acid, g) *cis*-aconitic acid, h) citric acid, i) DL-norleucine (IS), j) lactic acid, k) glutamine, l) glutamic acid, m) isocitrate, and n) pantothenic acid at MQC (20.0 μM).

For the optimum separation of analytes, several chromatographic parameters were investigated, including the He gas flow and injection volume. Based upon the findings, 1 ml/min He gas flow with 1.00 μl injection volume was chosen for further optimization based on peak shape and response. The efficiency of sample cleanup and chromatography can be demonstrated by flat baseline, with negligible influence of other endogenous components at the retention time of metabolites and IS (Fig 3).

The precision (%CV) system suitability test was with six consecutive injections of energy metabolite standards (20 μM) with IS (10μM). The reinjection reproducibility in the measurement of retention times for the analytes, expressed as % CV was ≤ 3.5 for 150 injections on the same column. The limit of detection (LOD) and LLOQ of the method were 0.01–0.03 and 0.05 μM for energy metabolites (Table 1).

The three calibration curves were linear over the concentration range 0.5–50 μM for TCA intermediates, with a correlation coefficient r^2^ ≥ 0.99 for all analytes. The accuracy and precision (%CV) for the calibration curve standard ranged from 87–108% and 2.1–8.9%, respectively. The intra-batch and inter-batch precision (%CV) varied 2.76–8.82 and the accuracy was within 91.5–111% (Table 3).

**Table 3:** Intra- and inter-day precision and accuracy of quality control samples for targeted TCA metabolites.

TCA metabolite (Nominal concentration)	Intra Quality Control Levels (Nominal Conc. (μM))											
	LLOQ QC (0.5 μM)			LQC (7.5 μM)			MQC (20.0 μM)			HQC (40.0 μM)		
	A	%CV	% Accuracy	A	%CV	% Accuracy	A	%CV	% Accuracy	A	%CV	% Accuracy
Pyruvic acid	0.52	5.54	105	7.91	4.85	106	21.6	8.23	108	44.4	8.54	111
Citric acid	0.48	7.71	95.7	7.57	6.35	101	19.8	2.68	99.2	42.0	7.04	105
*Cis*-aconitic acid	0.51	10.6	103	6.96	6.06	92.8	19.1	7.52	95.6	40.4	8.50	101
2-Oxoglutaric acid	0.52	8.59	104	7.07	8.03	94.3	19.7	9.46	98.7	39.4	6.10	98.5
Succinic acid	0.52	10.2	105	6.64	6.57	88.6	18.5	2.00	92.5	42.1	2.76	105
Fumaric acid	0.48	8.46	98.8	6.70	3.91	89.3	19.0	2.75	94.9	36.6	2.58	91.5
Malic acid	0.54	5.02	107	7.09	9.41	94.5	18.3	2.22	91.6	41.5	2.77	104
Oxaloacetic acid	0.52	8.79	104	7.23	7.19	96.5	19.4	5.24	97.2	38.1	3.22	95.2
TCA metabolite (Nominal concentration)	Inter Quality Control Levels (Nominal Conc. (μM))											
	LLOQ QC (0.5 μM)			LQC (7.5 μM)			MQC (20.0 μM)			HQC (40.0 μM)		
	A	%CV	% Accuracy	A	%CV	% Accuracy	A	%CV	% Accuracy	A	%CV	% Accuracy
Pyruvic acid	0.52	8.36	105	7.46	6.13	99.5	20.1	8.22	100	40.1	8.82	100
Citric acid	0.49	7.56	99.0	7.24	7.79	97.4	19.0	6.06	95.0	38.5	5.71	96.2
*Cis*-aconitic acid	0.53	8.48	106	7.03	7.52	93.7	18.9	8.38	94.6	38.1	6.25	95.2
2-Oxoglutaric acid	0.49	7.45	98.8	7.11	9.32	94.9	20.6	5.25	103	41.3	5.52	103
Succinic acid	0.48	8.75	96.4	6.83	8.12	91.1	19.7	8.27	98.7	39.7	5.06	99.3
Fumaric acid	0.54	6.51	107	6.80	7.17	90.7	19.1	6.29	95.5	37.7	3.85	94.2
Malic acid	0.53	7.94	106	7.45	5.70	99.4	18.3	3.72	91.5	38.3	4.15	95.3
Oxaloacetic acid	0.52	6.42	103	7.75	6.09	103	19.8	6.11	98.8	38.8	3.17	97.0

A: Mean concentration (μM), %CV: Percent co-efficient of variance, LLOQ QC: Lower limit of quantitation quality control

LQC: Low quality control, MQC: Middle quality control, HQC: High quality control.

Stock solutions kept for short periods (18 h) at room temperature and long-term storage for 20 days at 4°C, as well as freshly prepared solutions showed no evidence of degradation under all studied conditions. No significant degradation was observed for energy metabolites during sample storage and any of the processing steps during extraction. The detailed results for stability studies are presented in Table 4. The precision values for method ruggedness were 4.1–9.1%. The ability to dilute samples which could be above the upper limit of the calibration range was validated by analyzing six replicate samples containing 100 μM after five- to ten-fold dilution. The precision (% CV) values for dilution reliability were 4.6–9.3.

**Table 4:** Stability values for targeted TCA intermediates under different conditions.

Storage conditions		% Change (n = 6)							
	Level (μM)	Pyruvic acid	Citric acid	*Cis*-aconitic acid	2-Oxoglutaric acid	Succinic acid	Fumaric acid	Malic acid	Oxaloacetic acid
Process Sample Stability; 16h at 25°C	LQC	5.75	8.24	7.25	-4.61	6.94	9.73	6.82	4.36
	HQC	-3.06	3.61	8.92	0.23	12.90	8.02	-5.97	-6.79
Auto sampler Stability; 75h at 25°C	LQC	0.23	3.96	4.12	-3.24	0.68	6.15	4.48	3.06
	HQC	-0.41	2.65	6.71	5.35	9.76	8.20	0.61	-3.22
Dry Extract Stability; 32h at -70°C	LQC	-4.56	-4.84	-1.08	1.57	-4.90	9.32	-3.70	5.02
	HQC	-5.19	-0.88	7.81	6.07	10.32	8.73	5.77	3.68
Wet Extract Stability; 22h at 4°C	LQC	0.02	9.78	6.61	-6.33	0.71	-0.03	11.50	-0.06
	HQC	7.98	5.42	3.55	10.25	6.27	1.99	2.79	-5.88

% Change = (Mean stability samples–Mean comparison samples × 100)/ (Mean comparison samples).

n = Number of replicates for each level.

### Effect of high-fat diet on serum energy metabolites

**Fig 4:**
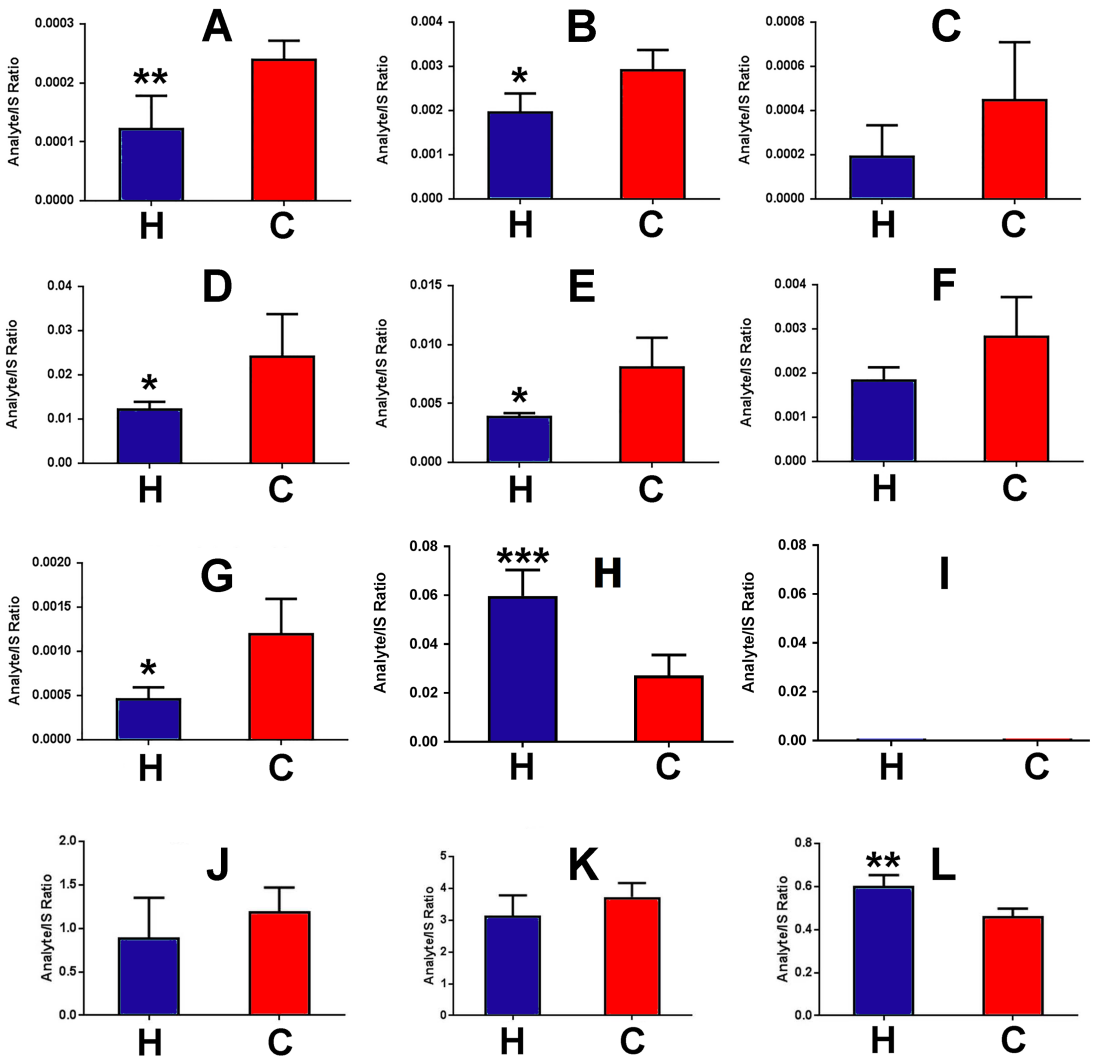
Changes for the serum energy metabolite/IS ratios for HFD (H)- and control chow diet (C)-fed mice (n = 5). A, pyruvic acid; B, citric acid; C, *cis*-aconitic acid; D, succinic acid; E, fumaric acid; F, malic acid; G, 2-oxoglutaric acid; H, glutamine; I, glutamic acid; J, lactic acid; K, isocitric acid; L, pantothenic acid. *p<0.05; **p<0.01.

Fig 4 shows analyte/IS ratios for 12 energy metabolites in mouse serum after HFD and control chow diet feeding for 8 weeks to 8-week-old C56BL/6N mice. HFD fed mice clearly had statistically significantly attenuated energy metabolites in serum, with pyruvate (Fig 4A; -49%), citrate (Fig 4B; -37%), succinate (Fig 4D; -50%), fumarate (Fig 4E; -50%), and 2-oxoglutarate (Fig 4G; -60%) serum concentrations all reduced. Serum glutamine (Fig 4H) and pantothenic acid (Fig 4L) were statistically significantly increased by 115% and 32%, respectively.

### Effect of high-fat diet on hepatic energy metabolites

**Fig 5:**
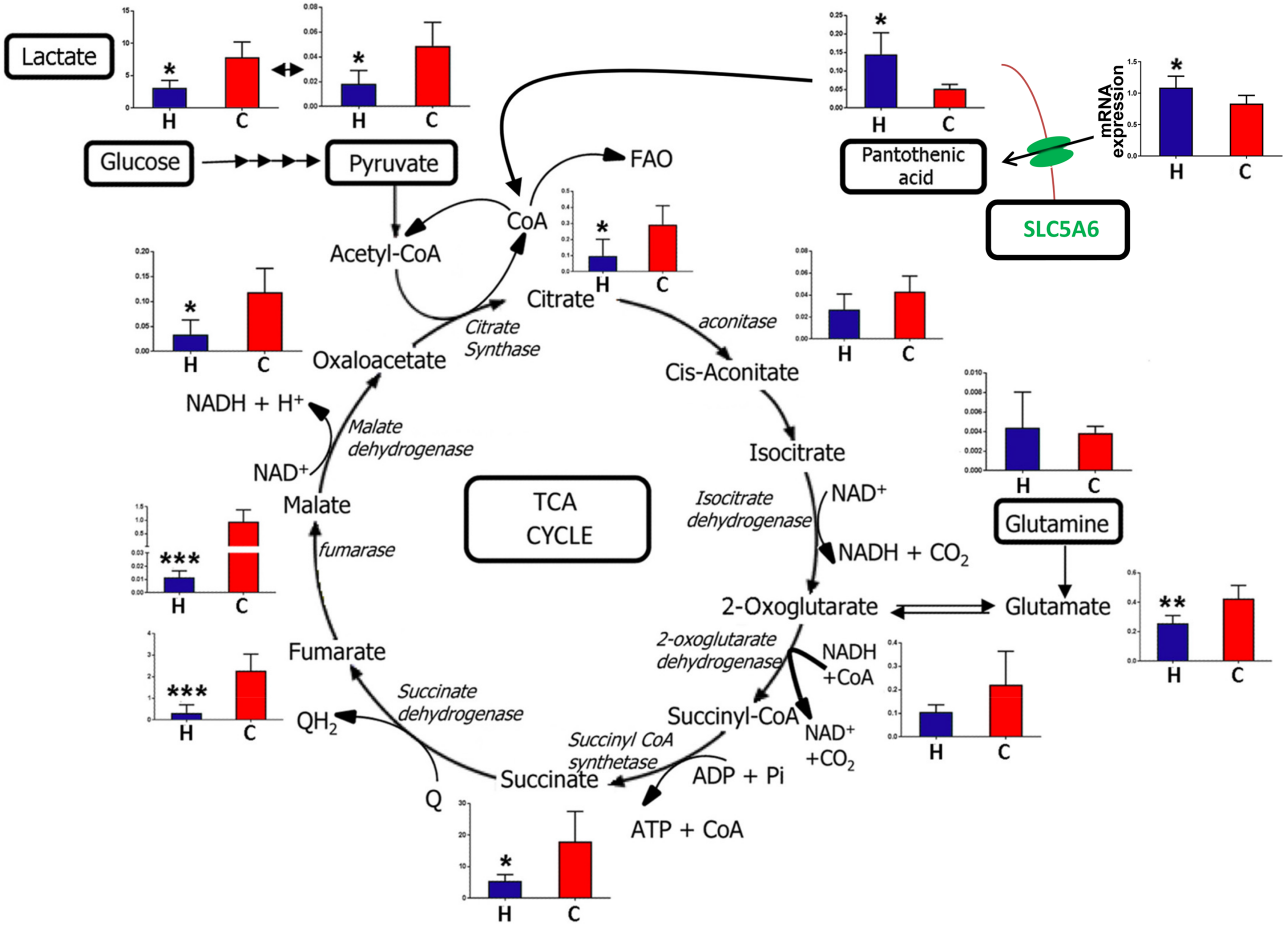
Representation of the TCA cycle and anaplerotic energy metabolites, showing metabolite levels after HFD (H) and control chow (C) feeding to 8 week-old mice for 8 weeks. Ordinate axes represent metabolite peak area/IS peak area, except for SLC5A6 expression (top right), where the ordinate represents mRNA expression.

Fig 5 represents a schematic of the TCA cycle, with the liver levels of each intermediate shown after HFD and control chow feeding for 8 weeks to 8-week-old C56BL/6N mice. In addition, levels for pyruvate and lactate are also shown, since pyruvate is generated from glucose by cytosolic glycolysis and enters the TCA cycle after conversion to acetyl-CoA. Glutamine is the second major energy source after glucose, entering the TCA cycle after conversion to glutamate by glutaminase and glutamate dehydrogenase to 2-oxoglutarate, As Fig 5 shows, hepatic glutamine concentration was not affected by HFD, but the concentration of resulting glutamate was impaired with HFD feeding. The findings shown in Fig 5 establish that HFD impairs hepatic cytosolic glycolysis with a 64% and 62% reduction in hepatic pyruvate and lactate concentrations, respectively. Interestingly, hepatic pantothenic acid concentration was elevated 175% with HFD. Pantothenic acid is a vitamin and an obligatory precursor foe Coenzyme A synthesis [42]. Pantothenic acid is imported to the cell by the sodium-dependent multivitamin transporter SLC5A6 [43]. After HFD feeding, hepatic *Slc5a6* gene expression was increased +27% (Fig 5).

The results show that the newly developed analytical method has the required sensitivity to characterize the altered levels of energy metabolites in liver and serum after feeding HFD and chow diet to mice for 8 weeks.

## Discussion

The ability to measure serum and hepatic levels of TCA and accessory metabolites using the newly developed assay demonstrates that HFD feeding to mice suppresses glycolysis, glutaminolysis and the TCA cycle. It is particularly notable that hepatic malate concentrations were suppressed 100-fold by HFD feeding for 8 weeks. This will affect the production of NADH by mitochondrial malate dehydrogenase. The malate-oxaloacetate shuttle serves to transport reducing equivalents produced by cytosolic glycolysis into the mitochondrion, since the mitochondrial inner membrane is impermeable to NADH. Malate produced from oxaloacetate + NADH in cytosol crosses into mitochondria where its reconversion to oxaloacetate generates NADH, which can be used for ATP generation by oxidative phosphorylation [44]. The results shown here suggest that the malate-oxaloacetate shuttle is also impaired under HFD feeding in mice. Hepatocytes would appear to have impaired energy production from glucose and glutamine under HFD feeding. It is possible that the liver generates energy from fatty acid β-oxidation (FAO), but this is an anaplerotic pathway that feeds the TCA cycle with acetyl-CoA. This process requires coenzyme A (CoA), which is synthesized from the vitamin pantothenic acid, and also cysteine and ATP [42]. As Fig 4 shows, hepatic pantothenic acid concentration was approx. three-fold enhanced after HFD feeding. Increases in both plasma and urinary pantothenic acid have been reported for rats fed a HFD [45]. The increase observed in this study may in part have been due to +27% increased expression of the *Slc5a6* gene encoding the pantothenic acid transporter SLC5A6. However, this was a meager increase compared to the much larger increase in hepatic pantothenic acid. It should be noted that the rate-limiting step in CoA synthesis is the initial 4'-phosphorylation of pantothenic acid by pantothenate kinase [42,46]. In isolated perfused rat hearts, pantothenate kinase was inhibited both by glucose and palmitic acid [46], both of which are expected to be elevated in the liver after HFD feeding. This perhaps better explains the +175% increase in hepatic pantothenic acid, rather than the smaller +27% increase in *Slc5a6* expression. Additionally, it should be noted that serum pantothenic acid was statistically significantly increased by +32%. If HFD induced hepatic *Slc5a6* expression, it should similarly have induced intestinal *Slc5a6* expression and therefore enhanced absorption of pantothenic acid from the diet. This is the most likely scenario given that the manufacturers' descriptions place pantothenic acid at 25 mg/kg for the NIH-31 pelleted chow but only 5.5 mg/kg for the HFD soft pellets. Thus, elevated serum pantothenic acid must have occurred secondary to massively increased absorption of this dietary vitamin.

Overall, HFD feeding impaired TCA cycle intermediates, glycolytic end-products, and glutamate, suggesting that anaplerosis was significantly decreased. In particular, the -68% decrease in hepatic citrate indicates that there was no increased anaplerotic flux of acetyl-CoA into the TCA cycle due to enhanced FAO. Nor was the liver of these animals using protein as an energy source because amino acids enter the TCA cycle after conversion to pyruvate (alanine, serine, glycine, threonine, cysteine, tryptophan), oxaloacetate (aspartate, asparagine), 2-oxoglutarate (glutamate, glutamine, proline, histidine, arginine), fumarate (phenylalanine, tyrosine), succinyl-CoA (methionine, isoleucine, valine), and acetyl-CoA (leucine, isoleucine, lysine, phenylalanine, tyrosine, tryptophan, threonine) [47]. None of these gateways would appear to be active after HFD feeding.

In conclusion, a highly reproducible SIM GCMS method was developed and for the simultaneous determination of energy metabolites after derivatization with MTBSTFA + 1% TBDMCS, both for serum and tissue samples. The method offers several advantages over reported procedures, in terms of sensitivity, lower sample requirements, a simple extraction procedure and overall analysis time. The efficiency of monophasic liquid extraction for liver tissue, protein precipitation for serum and a short chromatographic run time are highly favorable for high-throughput bioanalysis. Using this assay, analysis of serum and liver tissue from mice fed HFD and a control chow diet permitted insights into hepatic energy metabolism.

## Supporting information

S1 Fig Typical total ion chromatograms for serum extracts. a) high-fat diet fed mouse serum derivatized with MTBSTFA + 1% TBDMCS (see text), b) control chow fed mouse serum derivatized with MTBSTFA + 1% TBDMCS, c) high-fat diet fed mouse serum derivatized with BSTFA (see text), d) control chow fed mouse serum derivatized with BSTFA.
(TIF)

S2 Fig Typical total ion chromatograms for liver extracts. a) high-fat diet fed mouse liver derivatized with MTBSTFA + 1% TBDMCS, b) control chow fed mouse liver derivatized with MTBSTFA + 1% TBDMCS, c) high-fat diet fed mouse liver derivatized with BSTFA, d) control chow fed mouse liver derivatized with BSTFA.
(TIF)

S1 Table Published methods for the derivatization and quantitation of intermediary metabolites. (PDF)
