# Supplementary figures and images for: Metabolic profiling by gas chromatography-mass spectrometry of energy metabolism in high-fat diet-fed obese mice
Source: PLoS One. 2017 May 16;12(5):e0177953. doi: 10.1371/journal.pone.0177953 (PMC5433781; doi:10.1371/journal.pone.0177953)

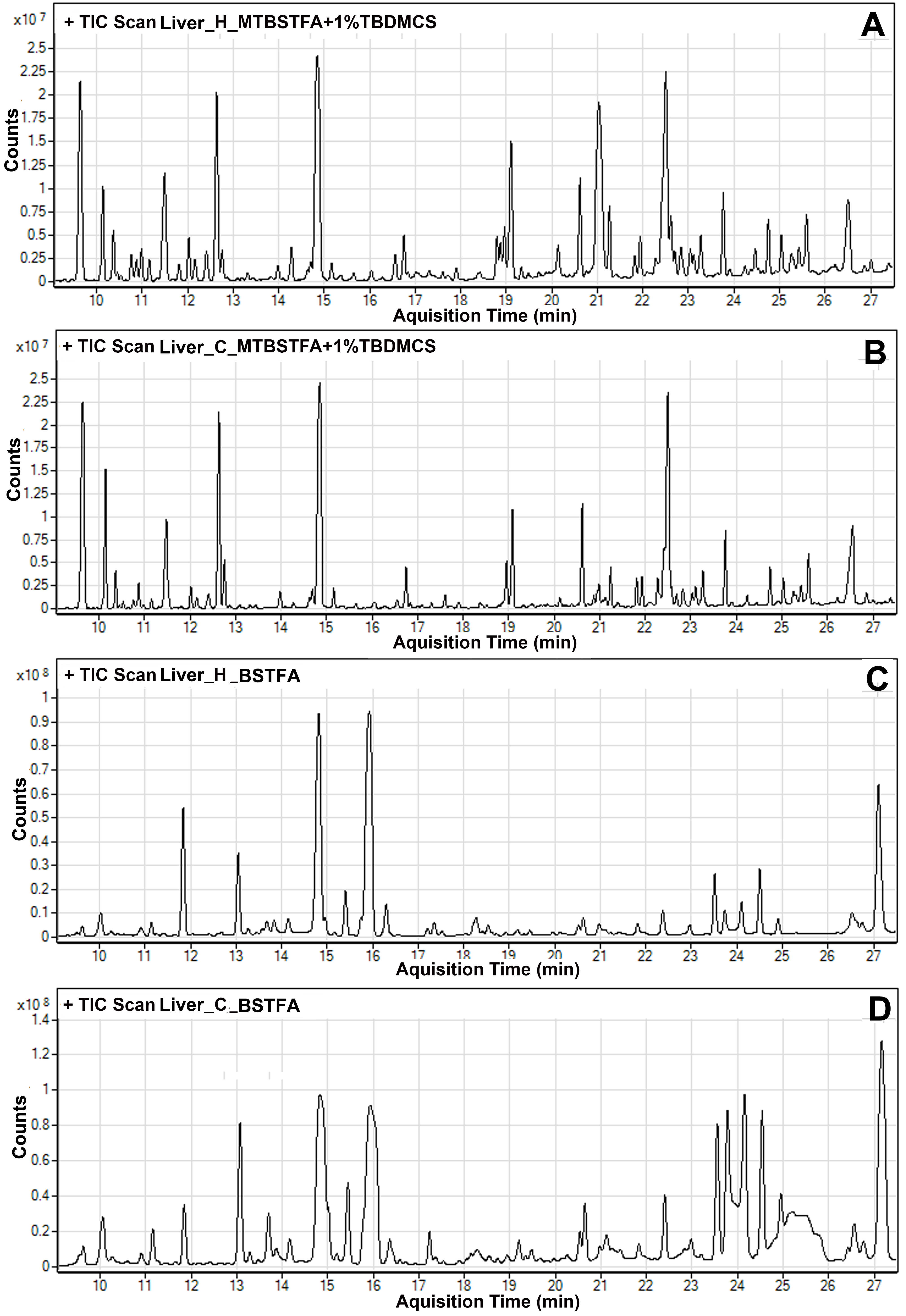

Supplement: S1 Fig — a) high-fat diet fed mouse serum derivatized with MTBSTFA + 1% TBDMCS (see text), b) control chow fed mouse serum derivatized with MTBSTFA + 1% TBDMCS, c) high-fat diet fed mouse serum derivatized with BSTFA (see text), d) control chow fed mouse serum derivatized with BSTFA. (TIF) [file pone.0177953.s001.tif]

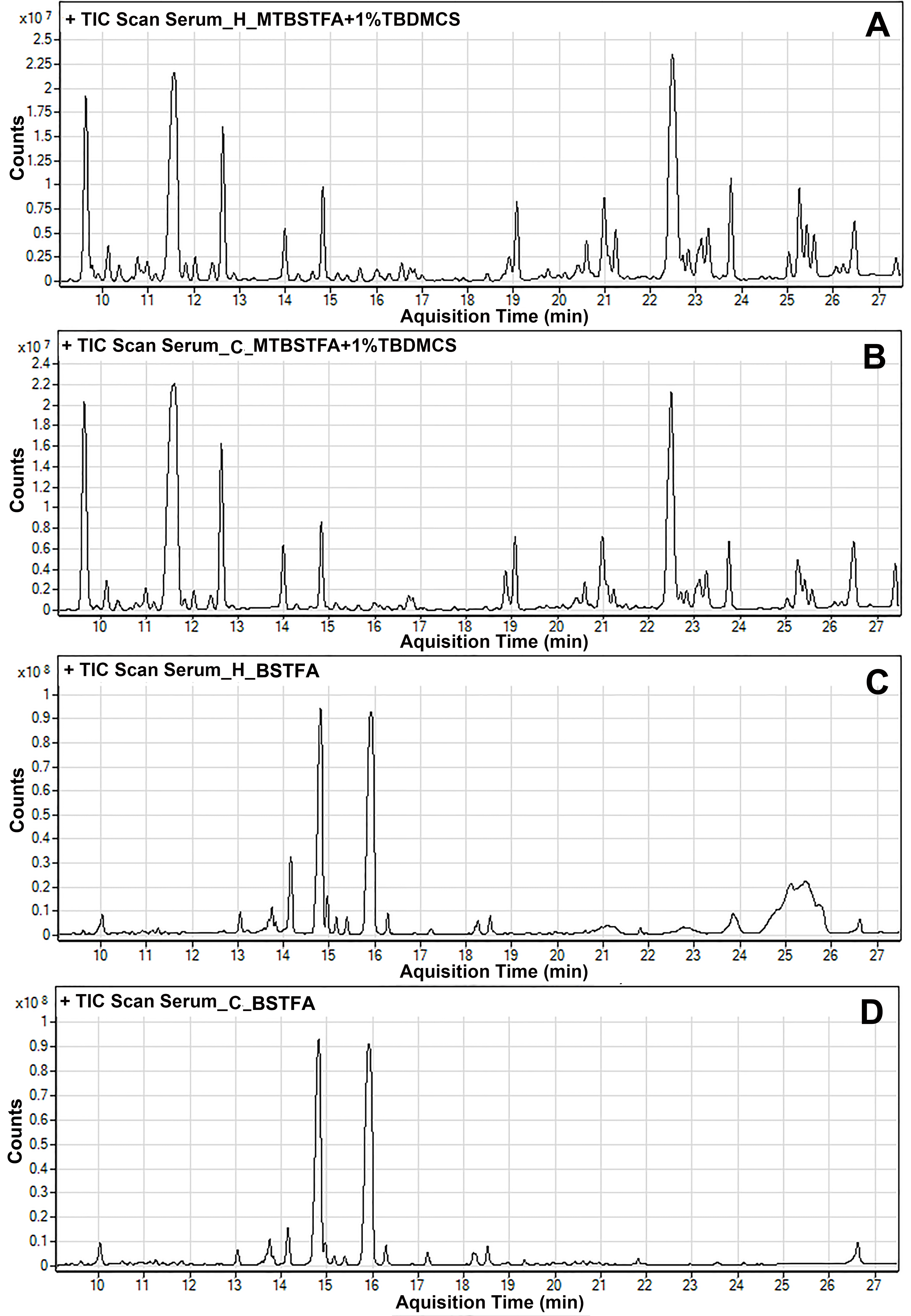

Supplement: S2 Fig — a) high-fat diet fed mouse liver derivatized with MTBSTFA + 1% TBDMCS, b) control chow fed mouse liver derivatized with MTBSTFA + 1% TBDMCS, c) high-fat diet fed mouse liver derivatized with BSTFA, d) control chow fed mouse liver derivatized with BSTFA. (TIF) [file pone.0177953.s002.tif]
