# Supplementary material for: Metabolic profiling by gas chromatography-mass spectrometry of energy metabolism in high-fat diet-fed obese mice
Source: PLoS One. 2017 May 16;12(5):e0177953. doi: 10.1371/journal.pone.0177953 (PMC5433781; doi:10.1371/journal.pone.0177953)
Supplement: S1 Table — (PDF) [file pone.0177953.s003.pdf]

S1 Table. Published methods for the derivatization and quantitation of intermediary metabolites

No.	Technique	Extraction procedure; Internal standard; Column	Linear range; Derivatization reagent	Application	Ref. no
1	GC-FID	Protein precipitation; glutaric acid; SE-30 packed column (2 m x 4 mm)	10-25 µg; *N*,*O*-bis-(trimethylsilyl)acetamide (BSA)	Insect hemolymph	32
2	LC-MS/MS	Liquid-liquid extraction; isotope-labeled internal standards; Waters XBridge C18 column (2.1 × 50 mm, Waters)	1.0-50 µg/mL; *O*-benzylhydroxylamine (OBH)	Cancer cells, tumor samples	33
3	GC-MS;	Protein Precipitation; [^{15}N]glutamic acid; FactorFour^{TM} VF-5ms capillary column (30 m × 0.25 mm, 0.25 µm)	2.0-20.0 µg/L; BSTFA + 1% TMCS[a]	Human blood	34
4	Ion-Exchange (IC-ED)	Monophasic liquid extraction; AS11-HC analytical column	Lactate (2-100 µM), malate (4-50 µM), phosphate (20-1000 µM), citrate (4-50 µM), *cis*-aconitate (2-50 µM)	CHO cells	35
5	GC-MS	Monophasic liquid extraction - liver tissue, protein precipitation-serum; DL-norleucine; capillary column (Agilent 30 m × 0.25 mm, 0.25 µm)	0.05-50 µM, MTBSTFA + 1% TBDMCS[a]	HFD treated fatty liver & serum and chow diet treated liver & serum	PW[b]

[a]for abbreviations, see text

[b]PW = present work
